# Downregulation of the CXCR4 receptor inhibits cervical carcinoma metastatic behavior *in vitro* and *in vivo*

**DOI:** 10.3892/ijo.2014.2383

**Published:** 2014-04-11

**Authors:** MAŁGORZATA SEKUŁA, KATARZYNA MIEKUS, MARCIN MAJKA

**Affiliations:** 1 Department of Transplantation, Polish-American Institute of Pediatrics, Faculty of Biochemistry, Biophysics and Biotechnology, Jagiellonian University, 30-663 Cracow, Poland; 2 Department of General Biochemistry, Faculty of Biochemistry, Biophysics and Biotechnology, Jagiellonian University, 30-663 Cracow, Poland

**Keywords:** cervical carcinoma, CXCR4, metastasis, tumor growth

## Abstract

Cervical carcinoma is frequently diagnosed among women, particularly in low and middle income countries. In this study, we investigated the role of the SDF-1/CXCR4 axis during cervical carcinoma growth and progression *in vitro* and *in vivo*. Downregulation of CXCR4 receptor using an RNA interference system led to almost complete inhibition of the receptor expression, activation and function. CXCR4 receptor silencing led to decreased ability to signal, to induce migration and to form holoclone-like colonies, with no influence on viability/proliferation of the cells. CXCR4-deficient cells had also significantly lower levels of MMP-9. Interestingly, downregulation of CXCR4 expression resulted in reduced tumor growth *in vivo*. Tumors generated by CXCR4-deficient cells had also lower expression of the proliferation marker Ki-67 and decreased ability to engraft into lungs and spleen. Taken together, our results indicate that CXCR4 receptor may play an important role during cervical carcinoma invasion. In our study CXCR4 influenced invasive properties of cervical carcinoma cells both *in vitro* and *in vivo*.

## Introduction

CXCR4 is a seven-span transmembrane G-protein coupled receptor for SDF-1 (stromal derived factor-1). The interaction between SDF-1 and its receptor CXCR4 activates multiple biological processes e.g. organogenesis and hematopoiesis, migration, proliferation, adhesion, inflammation and plays a critical role during tumor growth and metastasis (1–4). SDF-1 -CXCR4 axis has been shown to direct tumor cells to organs that highly express SDF-1 e.g., lymph nodes, lungs, liver or bones (5–7). It was also shown that cells expressing CXCR4 are clonally selected during their growth in the mammary fat pad of nude mice. A further increase in CXCR4 expression, and SDF-1-mediated migration was observed in cancer cells that metastasized to the lungs (8). Furthermore, blockade of CXCR4 inhibits tumor growth in a mouse model (9).

CXCR4 receptor is involved in a multistage process of metastasis (10,11). Scientific reports clearly indicate a significant role of CXCR4 receptor in breast, prostate, ovarian or melanoma cancer progression (12,13). In addition, CXCR4 tumor positive cells are characterized by high self-renewal abilities, tumor initiation and resistance to treatment (11,14). The ability of cancer cells to spread throughout the body is dependent on the interactions of their cell surface molecules with the microenvironment and the presence of cancer stem cells (CSCs) (11,15). CSCs initiate primary tumor growth, have the ability to self-renew and give rise to more differentiated cell types. They participate in cancer cell migration to distant tissue where they are able to form secondary tumor from a single cell (11,16). CSCs are very difficult to identify but recognition of them seems to be necessary for efficient cancer treatment. Their resistance to chemo- and radiotherapy is an additional exertion to elaborate relevant anticancer targeted therapy. Many of CSC markers are still unrecognized. Moreover, their expression profile may be addicted to the origin and type of the tumor. As previously described, CSCs demonstrate high expression of the CXCR4 receptor (17).

Cervical carcinoma (CC) is highly associated with human papillomavirus (HPV) infection that is one of the major risk factor for CC development. At early stages, CC cells occupy the surrounding tissue whereas in advanced stages they migrate and form metastases in regional lymph nodes, bone marrow, lungs, spleen and liver (18–20). However, despite the development of vaccine against HPV infection, cervical carcinoma is one of the most frequently diagnosed tumor among women with no available effective therapy at advanced stages.

Downregulation of gene expression is the appropriate method to evaluate the role of genes of interest (21,22). In our study we efficiently blocked CXCR4 gene expression with lentivirus shRNA construct directed against the CXCR4 gene and investigated the role of CXCR4 receptor in CC development and metastasis.

## Materials and methods

### Cell culture

HTB-35 cell line was purchased from ATCC (Rockville, MD, USA) and maintained in culture medium MEM (minimal essential medium, PAA Laboratories GmbH) supplemented with 10% heat-inactivated FBS (Fetal Bovine Serum, PAA) and 0.05 mg/ml gentamycin (PAA). Cells were cultured in a humidified atmosphere at 5% CO_2_ and 37°C. They were split usually twice a week.

### Lentiviral transduction

HTB-35 cells were transduced with Fusin shRNA Lentiviral Particles (Santa Cruz Biotechnology, Santa Cruz, CA, USA) in 6 *μ*g/ml polybrene (Sigma, St. Louis, MO, USA) according to the manufacturer’s protocol. Transduced cells were selected with 2.5 *μ*g/ml puromycin (InvivoGen, San Diego, CA, USA) for 16 days. Next, the cell line was analyzed to confirm the reduction of CXCR4 gene expression at the mRNA and protein level and subjected to further characterization. For control cell line, HTB-35 cells were transduced with Control shRNA Lentiviral Particles (Santa Cruz Biotechnology) as described above.

### Flow cytometry

Extracellular staining of CXCR4 receptor was tested by flow cytometry. Cells were split with nonenzymatic Cell Dissociation Solution (Sigma). Cells (1×10^5^) in 90 *μ*l staining buffer (PBS supplemented with 2% FBS) were added to a test tube containing the appropriate amount of monoclonal PE-conjugated mouse anti-human CD184 antibody (clone 12G5, BD Bioscience-Pharmingen, San Jose, CA, USA). Isotype-matched mouse PE-conjugated immunoglobulin (IgG2aκ) served as control (clone G155-178, BD Pharmingen, San Jose, CA, USA). After 30 min of incubation on ice in the dark, cells were washed with staining buffer twice and collected using a FACSCanto cytometer (Becton-Dickinson, San Jose, CA, USA). Data were analyzed with FACS Diva (Becton-Dickinson), WIN MDI 2.9 (free program available) and Cyflogic v.1.2.1. software (free program available).

### Quantitative real-time PCR (qRT-PCR) analysis

The total RNA isolation was performed using RNeasy Mini kit (Qiagen, Hilden, Germany). followed by DNAse treatment (Promega, Madison, WI, USA). RNA (1 *μ*g) was used for the reverse transcriptase reaction that was carried out using M-MLV reverse transcriptase (Promega) according to the manufacturer’s protocol. qRT-PCR analysis was performed on ABI PRISM 7300 Sequence Detection System (Applied Biosystems, Inc., Foster City, CA, USA) using TaqMan Gene Expression Master MiX (Applied Biosystems, Inc.). Probes used in this study were as follows: human GAPDH (Hs99999905_m1), MMP9 (Hs00234579_m1), TIMP1 (Hs00171558_m1), TIMP2 (Hs00234278_m1), HIF-1α (Hs00153153_m1), VEGF (Hs00900055_m1) and mouse GAPDH (Mm99999915_g1). The mRNA expression level was normalized to the housekeeping gene GAPDH. The experiments were performed three times in duplicate. Data are presented as % of control cells (wild-type).

### MTS assay

To examine mitochondrial activity of tumor cells MTS test was done. Cells (2×10^3^) were seeded on 96-well plates. Analysis was performed in two different conditions: culture medium supplemented with FBS (MEM 10% FBS) and culture medium supplemented with bovine serum albumin (MEM 0.5% BSA). After 24, 48, 72 and 96 h CellTiter 96^®^ AQueous One Solution assay (Promega) was added according to the manufacturer’s protocol. After 2 h the level of absorbance was read at a wavelength of 490 nm using the EL×800 Universal Microplate Reader (BioTek Instruments, Highland Park, USA) and analyzed with KC4 v3.0 with PowerReports software. The results are presented as mean absorbance value in an appropriate time. The experiment was carried out three times in triplicates.

### Colony-forming assay

To determine different colony morphologies, 1×10^3^ cells were plated as a single cell on a 6-well plate in culture medium. As a colony, we recognized a cluster consisting of at least 6 cells. After 4 days different colony morphologies were observed. Paraclone-, holoclone- and meroclone-like colonies were identified. Colonies were fixed, stained using Wright’s reagent (Merck, Darmstadt, Germany) and counted in 10 fields at ×100 magnification under a light Olympus BX-51 microscope. Two independent experiments were performed. The percentage of paraclone- and holoclone-like colonies recognized as a result of cell culture in low density ± D are presented.

### Suspension growth assay

Cells were seeded at a density of 3×10^4^ on a non-adherent 24-well plate (Thermo Scientific, Rockford, IL, USA) in culture medium for 48 h at 37°C and 5% CO_2_. After this time cells were collected and seeded at 1×10^3^ on a 6-well plate in order to observe the clonal growth potential and in the amount of 2×10^3^ on a 96-well plate to determine the mitochondrial activity potential (MTS assay). Experiments were performed two times according to the schemes described above.

### Cell stimulation

Cells (2.5×10^5^) were seeded on 6-well plates in culture medium. The next day, culture medium was changed to MEM 0.5% BSA to make cells quiescent. Stimulation was performed with SDF-1β (100 ng/ml) (PeproTech, Rocky Hill, NJ, USA) for 2, 5, 10 and 30 min. Medium containing 10% FBS and 0.5% BSA were positive and negative control, respectively.

### Western blot analysis

Cells were lysed (for 10 min) on ice in M-Per lysing buffer (Pierce) containing protease and phosphatase inhibitors (Sigma). Protein concentration was determined by Bradford protein assay. Protein samples [containing 20 μg of protein, LDS (NuPage LDS sample buffer; Invitrogen Life Technologies, Carlsbad, CA, USA), Bond Breaker (Thermo Scientific) and M-Per buffer] were separated on a 12% sodium dodecyl sulfate-PAGE gel and transferred into a PVDF membrane (Bio-Rad Laboratories, Hercules, CA, USA). The phosphorylation of AKT and MAPK was assessed using primary rabbit anti-phospho-AKT (Ser 473, Cell Signaling, Danvers, MA, USA) and primary mouse anti-phospho-MAPK (Thr202/Tyr204, Cell Signaling) antibodies and subsequently detected with horseradish peroxidase (HRP)-conjugated goat anti-rabbit IgG secondary antibody (sc-2054; Santa Cruz Biotechnology) and (HRP)-conjugated goat anti-mouse IgG secondary antibody (sc-2055; Santa Cruz Biotechnology). The membranes were developed with an enhanced chemiluminescence reagent (ECL, Amersham Life Sciences, Buckinghamshire, UK) dried and subsequently exposed to the HyperFilm (Amersham Life Sciences) or imaged by Gel Logic Imaging System 1500 (Kodak; Molecular Imaging System, New Haven, CT, USA). An equal loading in the lanes was evaluated by probing with anti-rabbit monoclonal anti-GAPDH antibody (14C10; Cell Signaling). The experiment was performed two times with similar results. Representative data are presented.

### Chemotaxis assay

To check the tumor cells the migration ability towards SDF-1β gradient, modified Boyden’s chamber with 8-*μ*m pore polycarbonate membrane inserts (Costar Transwell; Costar-Corning, Lowell, MA, USA) were used. Cells were harvested with non-enzymatic Cell Dissociation Solution (Sigma). Cell suspension at the density of 3×10^4^ in 100 *μ*l MEM 0.5% BSA was placed in the upper chamber of the insert. In the lower chamber 650 *μ*l medium contained SDF-1β [100 ng/ml] was placed. After 24-h incubation in a humidified atmosphere at 5% CO_2_ and 37°C the transmigrated cells were fixed, stained with Wright solution (Merck) and counted in five fields of view at ×100 magnification using an inverted light microscope (Olympus IX70). Medium containing 10% FBS, or 0.5% BSA was the positive and negative control, respectively. Experiment was performed two times in duplicates.

### In vivo tumor models (animal experiments)

The 6- to 8-week old male NOD-SCID mice (non-obese diabetes severe combined immunodeficiency mice) were used to evaluate the *in vivo* meta-static behavior of tumor cells.

Subcutaneous injection: mice were injected with 5×10^6^ tumor cells/mouse. Twice a week the volume of primary tumors was quantified using the formula [(x^2^y)/2] for an ellipsoid. After 30 days mice were subjected to anesthesia, tumors were weighed and fixed in 10% formalin. Immunohistochemical evaluation was prepared using hematoxylin and eosin staining and primary mouse monoclonal antibodies anti-Ki-67 (clon MIB-1; 1:75, Biocompare, DakoCytomation, Glostrup, Denmark).

Intravenous injection: mice were injected through the eyeball with 1×10^6^ tumor cells per mouse for 24 h and 30 days. After this time, mice were sacrificed. Isolation of bone marrow was performed. Organ tissues such us lungs and spleen was isolated and homogenized using Cell Strainer (BD Bioscience) with a 40-micron pore size. To assess the potential sites of metastasis, RNA isolation, reverse transcription and qPCR (as previously described) was performed to define human to mouse GAPDH proportion.

Animal experiments were approved by the Local Ethics Committee for Experiments on Animals acting at the Jagiellonian University in Krakow (Resolution No. 56/2011). Two independent experiments were carried out with 10 NOD-SCID mice/group.

### Statistical analysis

Statistical analysis was performed using Statistica v10 software by one-way ANOVA and the Tukey test. The results with P-values <0.05 were considered as statistically significant, and labeled by an asterisk in the figures.

## Results

### Downregulation of CXCR4 gene expression

**Figure 1.**
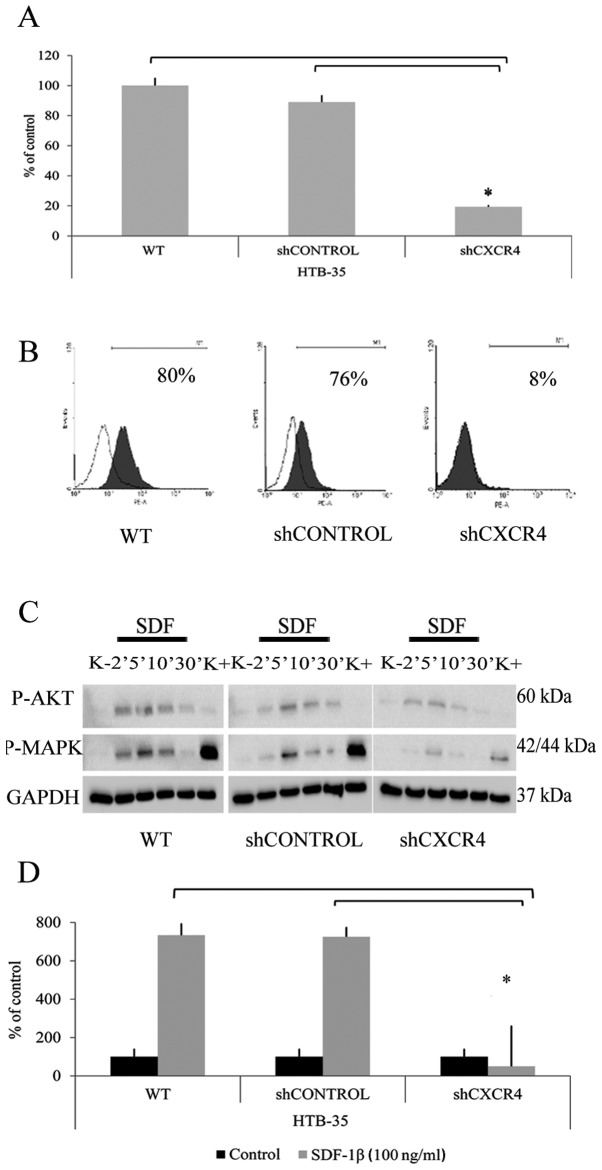
The confirmation of CXCR4 gene downregulation. Transduction with fusin shRNA lentiviral particles obtained 80% reduction of CXCR4 expression at mRNA level [(A) real-time PCR] and 90% reduction of CXCR4 at protein level [(B) FACS analysis]. (C) The expression of phospho-AKT and phospho-MAPK after human SDF-1β (100 ng/ml) stimulation of control cells (HTB-35 WT and HTB-35 shCONTROL) and examined the cells (HTB-35 shCXCR4) at 2, 5, 10 and 30 min. Strong phosphorylation of AKT and MAP kinases after 5 min stimulation in control cells was observed. CXCR4 receptor downregulation resulted in lower phosphorylation level of the examined pathways. Medium containing 10% FBS or 0.5% BSA consisted of positive and negative control, respectively. GAPDH was used as a control of equal loading. The experiment was performed two times with similar results. Representative data are presented. (D) The significant reduction of shCXCR4 cell migration potential towards SDF-1β gradient (100 ng/ml) compared to control cells (HTB-35 WT and HTB-35 shCONTROL) was observed in chemotaxis assay; ^*^P<0.05, ±SD.

In order to efficiently knock down the CXCR4 gene expression, HTB-35 cell line was transduced with Fusin shRNA lentiviral particles, and shRNA lentiviral particles were used as a control. After transduction and antibiotic selection, we obtained 80% and 90% reduction of CXCR4 gene expression at mRNA and protein level, respectively, compared to control cells: wild-type (WT) and shCONTROL (Fig. 1A and B).

Next, we examined the effectiveness of the CXCR4 gene knockdown. Western blot analysis showed strong phosphorylation of AKT and MAPK kinases after 5 min stimulation in control cells. The shCXCR4 cells also responded to the chemokine but at a lower level. The weak stimulation might be caused by CXCR7 receptor activity, the second SDF-1 receptor (Fig. 1C). Moreover, downregulation of CXCR4 receptor led to almost 7-fold decrease in the chemotactic activity toward SDF-1β gradient compared to control cells (Fig. 1D).

### CXCR4 receptor maintains the diversity of clonal morphology

**Figure 2.**
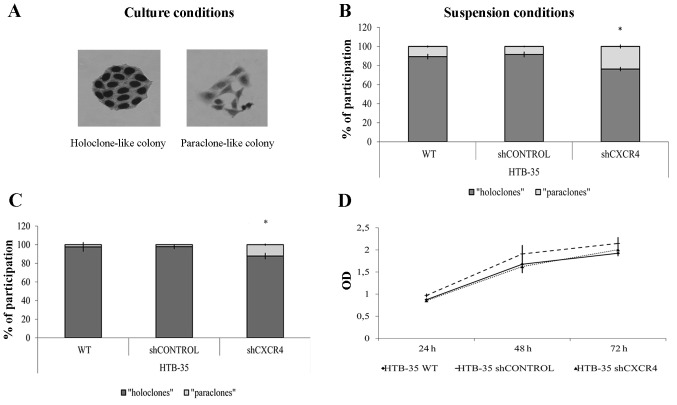
Colony-forming assay resulted in diversity of clonal morphology after culture at low density. (A) Colony morphology was observed as holoclone-like colony structures with stem cell characteristic and paraclone-like colony structures formed by differentiated cells. (B) After 48-h growth at suspension condition mitochondrial activity was checked. We observed no differences in MTS assay between examined and control cells (B). Culture conditions (C) and suspension conditions (D) lead to increased number of paraclone-like colonies depending on the decrease of CXCR4 level. The experiment was carried out two times in triplicates. Data are presented as mean optical density (OD) ± SD at the appropriate time.

The epithelial origin of HTB-35 cell line is associated with the capacity to form colony-like structures as a result of culture beginning at low density. In order to analyze whether the CXCR4 receptor is involved in the diversity of clonal morphology, colony-forming assay was used. After 6-day culture at low density, ‘holoclone’-, ‘meroclone’- and ‘paraclone’-like colonies were identified (Fig. 2A). Our results suggest that CXCR4 receptor downregulation increases the number of ‘paraclone’-like colonies in comparison to control cells (Fig. 2B). Cell culture under the suspension condition for 48 h has no influence on the colony formation but changes the percentage participation of different types of colonies. Control cell lines had lost cells which are able to form ‘paraclone’-like colonies. Similar effect was observed in shCXCR4 cells where the percentage of ‘paraclone’-like colonies decreased about 50% compared to control condition (Fig. 2C). Furthermore, growth in suspension has no influence on mitochondrial activity of examined cells (Fig. 2D).

### CXCR4 receptor modulates the expression of MMP-9

**Figure 3.**
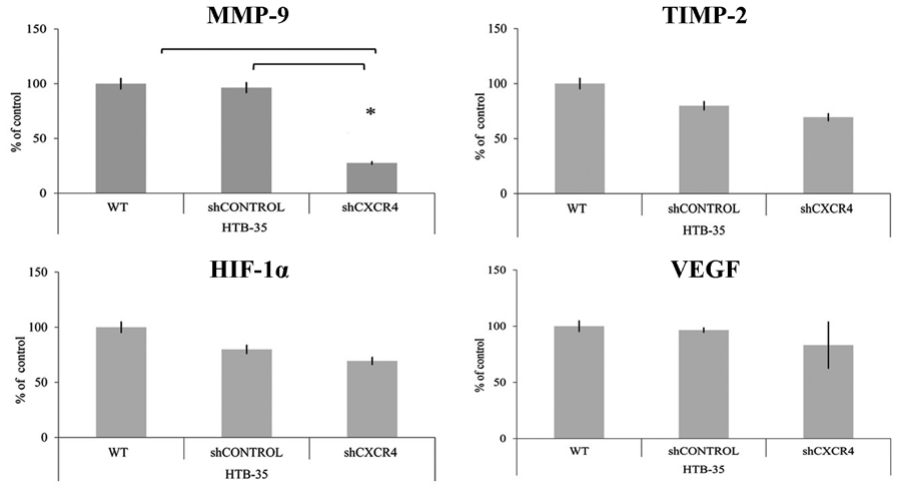
The influence of CXCR4 gene downregulation of the expression of genes related with metastasis and angiogenesis at mRNA level. Reduced level of MMP-9 was observed in shCXCR4 cell line. The TIMP-2, HIF-1α and VEGF levels were not changed according to CXCR4 gene expression. The mRNA expression level was normalized to the human housekeeping gene GAPDH. The experiments were performed two times in duplicate. Data are present as % of control cells (WT, wild-type).

To evaluate if CXCR4 receptor mediates the expression of genes related to angiogenesis and metastasis, quantitative real-time RT-PCR was performed. We observed significant positive correlation between CXCR4 and matrix metalloproteinase (MMP-9) level. CXCR4 downregulation resulted in the reduction of MMP-9 and had no influence on tissue matrix metalloproteinase inhibitor-2 (TIMP-2) expression. In HTB-35 cell line, the interaction at mRNA level between CXCR4 and hypoxia-inducible factor 1-α (HIF-1α) or vascular endothelial growth factor (VEGF) was not observed (Fig. 3).

### CXCR4 downregulation does not influence the cell proliferation rate in vitro but efficiently reduces tumor growth and metastasis in a murine model in vivo

**Figure 4.**
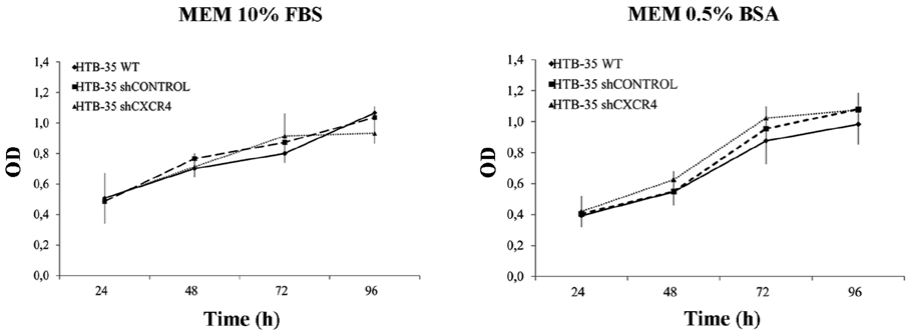
The influence of CXCR4 gene downregulation on mitochondrial activity of HTB-35 cell line *in vitro*. Cells were cultured in medium supplemented with 10% FBS or 0.5% BSA. MTS test was performed at 24, 48, 72 and 96 h. We observed no differences between the examined cell lines regardless of standard or starvation condition. The experiment was carried out three times in triplicates. Data are presented as mean optical density (OD) ± SD at the appropriate time.

The effect of CXCR4 downregulation on HTB-35 tumor cell proliferation rate was measured by MTS assay. Cells were cultured for 96 h in medium supplemented with 0.5% BSA or 10% FBS. We observed no differences in the proliferation rate between shCXCR4 cells and control cells either under starvation or control conditions (Fig. 4).

**Figure 5.**
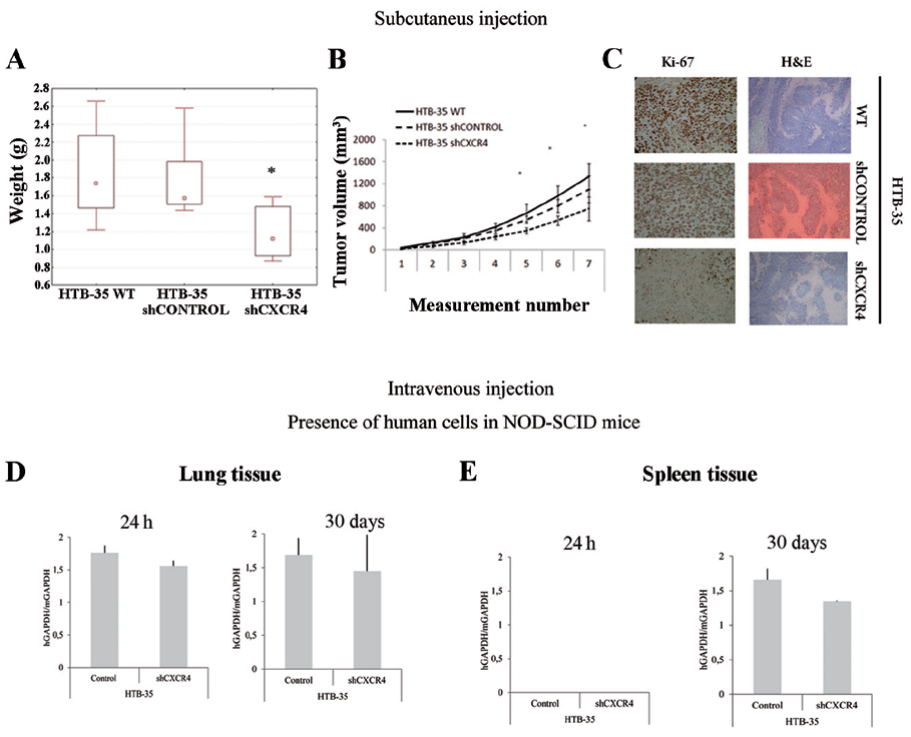
The influence of CXCR4 gene downregulation on tumor growth *in vivo* in a murine model. Subcutaneus injection of 5×106 cells/mouse resulted in reduced tumor weight (A) and proliferation potential (B) in shCXCR4 cells compared to control cells (WT and shCONTROL). The reduction of Ki-67 antigen, as a prolifer ation marker, was detected in shCXCR4 primary tumor tissue with no simultaneous changes in H&E staining (C). Intravenous injection of 1×10^6^ cells/ mouse resulted in decreased hGAPDH/mGAPDH ratio in lung tisssue in short- and long-term murine models of about 12 and 20%, respectively in shCXCR4 group compared to control group (D). Time of 24 h was not sufficient to populate the spleen tissue of tumor cells, but after 30 days, 30% reduction of hGAPDH/ mGAPDH ratio was associated with CXCR4 downregulation (E). The experiment was performed two times. The total number of mice was 10 per each group.

Interestingly, the results from *in vitro* tests were different from *in vivo* results. Our studies showed that CXCR4 down-regulation reduces HTB-35 cell growth and metastasis. Subcutaneous injection with shCXCR4 cells in NOD-SCID mice was associated with a significant decrease (about 30%) in tumor growth potential (Fig. 5A and B) compared to control cells. In addition, Ki-67 staining showed decreased proliferation activity in tumors formed by shCXCR4 cells (Fig. 5C). However, H&E staining revealed no differences between morphology of the tumors formed by shCXCR4 cells and control cells (Fig. 5C).

Intravenous injection demonstrated the participation of SDF-1/CXCR4 axis in CC cell migration to the lungs and spleen. High expression of human GAPDH in lungs tissue in both control groups of NOD-SCID mice was observed. In contrast, CXCR4 receptor downregulation decreased the metastatic ability of HTB-35 cells. We observed 12% and 20% decrease in metastasis in short- and long-term murine models, respectively (Fig. 5D). We also showed that the period of 24 h was not sufficient for tumor cells to extravasate into spleen tissue. After 30 days of injection, we observed 30% reduction in metastasis for shCXCR4 in comparison to control group (Fig. 5E). We detected no human cells in the bone marrow after injection with HTB-35 cells (data not shown).

## Discussion

Growth disorders usually manifest by excessive proliferation which cause e.g., rapid primary tumor growth and subsequently its invasion and metastasis (23). The role of the chemokine receptor CXCR4 in the regulation of tumor growth has been recognized as an important issue. In HTB-35 cell line, CXCR4 downregulation did not change the mitochondrial activity and growth potential *in vitro*. Interestingly, we observed a significant decrease in primary tumor growth and weight *in vivo*. It is possible that the CXCR4 receptor is required for the initiation of cell proliferation and/or promotes the survival of cervical cancer cells, similarly to breast cancer cells (24). The observed effect of limiting growth *in vivo* might be explained by low expression of CXCR4 which is associated with induction of apoptosis and/or a decrease level of proliferation potential (24). In addition, Ki-67 staining was performed to check the proliferation activity of tumor cells *in vivo*. Ki-67 antigen is expressed from late G1 to the M phase. It has a prognostic value and its high level is associated with poor prognosis and short patient survival (25). In this study, we observed that a decrease of proliferation activity in primary tumors formed by shCXCR4 cells, was associated with the reduction of Ki-67 level. Similar data were reported for breast and lung cancer, where increased growth was directly related to Ki-67 upregulation and provides enhanced risk of metastasis (26,27).

Our data define the CXCR4 receptor as a very important factor in the metastatic process of cervical cancer. Morphological diversity is a common feature of cancer cells and might be associated with the ability to generate colonies and self-renewal population from single cell (11,28–30). The study of CSCs and the mechanisms that regulate their biology is challenging. CSCs constitute a small percentage of the tumor population. However, we are still unable to define precisely markers of these cells for many types of tumors. One possible pre-selection of epithelial cells as stem cells is to assess their clonogenic potential (11,28). Such cells are capable of forming specific clones of different morphology which is an indicator of their properties (29). There are: i) holoclones, the structure typical for epithelial cells, composed of small, closely adjacent cells with increased capacity for self-renewal and differentiation properties, ii) paraclones, the structure formed by the cells with irregular shape, loosely arranged in the clone with a high degree of differentiation capacity and iii) meroclones, intermediate forms of clones (28,30). Among holoclones, there is the greatest probability of stem cell presence [e.g. keratinocytes (31)] or CSCs [e.g. prostate cancer (29)]. Only cells derived from holoclones are able to initiate tumor development after transplantation into mice. Furthermore, they express surface markers characteristic of CSCs (such as CXCR4 and CD44). Interestingly, meroclone-derived cells are not able to form tumor tissue. Furthermore, paraclone-derived cells die during the *in vitro* culture (29) which was also observed in our study in suspension culture. Our results indicate that CXCR4/SDF-1 axis play a very important role in maintaining the metastatic potential. Morphological diversity showed that CXCR4 downregulation caused the reduction of ‘holoclone’-like structures possibly indicating a decreased number of CSCs. However, further analysis and characterization of colonies formed by HTB-35 cells are required, but these results reflect a decreased metastatic potential to the lung and spleen tissue in the shCXCR4 cell line. Our results are consistent with a study of human breast cancer that also demonstrated the importance of CXCR4/SDF-1 signaling at the primary tumor microenvironment (32). It is possible that SDF-1 in the tumor microenvironment promotes breast cancer proliferation, migration and invasion. The impairment of CXCR4 and/or SDF-1 activity could interrupt this paracrine signaling pathway reducing growth potential of primary tumors (24,32). As previously described, CXCR4 receptor is also related to bone marrow metastasis in e.g. rhabdomyosarcoma (33), and breast cancer (34). Our observation indicates the attenuation of AKT and MAPK pathways and inhibition of chemotaxis to the SDF-1 gradient in shCXCR4 cells. It may suggest a similar mechanism of CXCR4/SDF-1 axis in the cervical cancer cells as in the breast cancer and rhabdomyosarcoma (9,24,33).

High level of MMP expression is directly associated with tumor invasiveness and poor prognosis (35–37). We observed positive relationship between CXCR4 and MMP-9 level in HTB-35 cell line. Significant decrease of MMP-9 mRNA expression level in the shCXCR4 cells might be associated with reduced capacity to form lung and spleen metastasis. Surprisingly, the level of HIF-1α and VEGF was not changed. The obtained results are different from studies of breast cancer angiogenesis. It has been showed that HIF-1α is a potent inducer of VEGF but the expression of VEGF can be unregulated via HIF-1α independent mechanisms as well (38). It is related to the activation of PI3K/AKT pathway, which can be stimulated, for example, by CXCR4/SDF-1 interaction. The inhibition of this axis significantly decreases the VEGF expression and angiogenesis in MDA-MB-231 cells (39).

Inhibition of CXCR4 expression and function significantly impairs the growth potential of the HTB-35 cervical carcinoma cell line *in vivo* and efficiently decreases the lung and spleen metastasis in an animal NOD-SCID model. Thus, our data suggest CXCR4 as a novel target for prevention of cervical carcinoma growth and metastasis.
